# Four-dimensional flow MRI of stented versus stentless aortic valve bioprostheses

**DOI:** 10.1007/s00330-017-4953-2

**Published:** 2017-07-14

**Authors:** Floortje van Kesteren, Laurens W. Wollersheim, Jan Baan, Aart. J. Nederveen, Abdullah Kaya, S. Matthijs Boekholdt, Bas A. de Mol, Pim van Ooij, R. Nils Planken

**Affiliations:** 1 Department of Radiology and Nuclear Medicine, Academic Medical Centre, University of Amsterdam, Meibergdreef 9, 1105 AZ Amsterdam, The Netherlands; 2 Department of Cardiology, Academic Medical Centre, University of Amsterdam, Amsterdam, The Netherlands; 3 Department of Cardiothoracic Surgery, Academic Medical Centre, University of Amsterdam, Amsterdam, The Netherlands

**Keywords:** Four-dimensional MRI, Cardiovascular magnetic resonance, Heart valve prosthesis, Stented, Stentless

## Abstract

**Objectives:**

To evaluate aortic velocity, wall shear stress (WSS) and viscous energy loss (EL) of stented and stentless bioprostheses using 4D flow MRI 1 year after surgical aortic valve replacement.

**Methods:**

For this cross-sectional study 28 patients with stented (n = 14) or stentless (n = 14) bioprosthesis underwent non-contrast-enhanced 4D-flow MRI at 1.5 T. Analyses included a comparison of velocity, WSS and EL in the ascending aorta during peak systole for both spatially averaged values and a comparison of local differences using per-voxel analysis.

**Results:**

No significant differences were found in peak and mean velocity (stented vs. stentless: 2.45 m/s vs. 2.11 m/s; p = 0.09 and 0.60 m/s vs. 0.62 m/s; p = 0.89), WSS (0.60 Pa vs. 0.59 Pa; p = 0.55) and EL (10.17 mW vs. 7.82 mW; p = 0.10). Per-voxel analysis revealed significantly higher central lumen velocity, and lower outer lumen velocity, WSS and EL for stentless versus stented prostheses.

**Conclusion:**

One year after aortic valve implantation with stented and stentless bioprostheses, velocity, WSS and EL were comparable when assessed for averaged values in the ascending aorta. However, the flow profile described with local analysis for stentless prosthesis is potentially favourable with a significantly higher central velocity profile and lower values for outer lumen velocity, WSS and EL.

***Key Points*:**

• *Stentless bioprostheses can be implanted instead of stented aortic valve bioprostheses*.

• *Haemodynamic performance of valve prosthesis can be assessed using 4D flow MRI*.

• *Averaged ascending aorta PSV*, *WSS and EL are comparable 1 year post*-*implantation*.

• *Centreline velocity is highest*, *WSS and EL is lowest for stentless prosthesis*.

## Introduction

Surgical aortic valve replacement is the standard treatment for patients with advanced aortic valve disease including severe and symptomatic aortic valve stenosis [1, 2]. Nowadays, a bioprosthetic heart valve is used in more than three-quarters of surgical aortic valve replacements [3]. Traditional bioprosthetic valves have a stented framework made of metal, with valve leaflets mounted on the stent to resemble a native tri-leaflet valve. Recently, bioprostheses have become available that replaced metal stents with polymer stents. Although the stented design facilitates easy implantation, it reduces the effective orifice area and obstructs laminar blood flow [4]. As an alternative, stentless bioprostheses have been introduced. Because of the absence of a space-consuming stent, stentless bioprostheses should improve haemodynamic performance compared to stented bioprostheses. Indeed, transthoracic echocardiography observations revealed lower valvular pressure gradients in stentless valves compared to stented valves [5]. In addition, without obstruction due to space-consuming stents, flow profiles are expected to show lower flow velocities, lower wall shear stress (WSS) and less viscous energy loss (EL). WSS is the tangential force of blood flow on the endothelial cells outlining the vessel wall. High aortic WSS can occur for example by obstructed flow and may lead to aortic dilation [6, 7]. Furthermore, flow obstruction causes energy loss and viscous EL can be used as a measure of aortic blood flow disturbance [8].

Current evidence for a better haemodynamic performance of stentless bioprostheses is limited. Recently, four-dimensional (4D) flow magnetic resonance imaging (MRI) (time-resolved three-dimensional (3D) phase contrast imaging, with velocity encoding in all principal velocity directions) has become available. This technique improves the understanding of blood flow patterns through the heart and large vessels [9, 10]. In addition to echocardiography, 4D flow MRI measurements can be used for visualisation and quantification of blood flow volumes and flow profiles [11]. The aim of this study was to compare the performance of stented and stentless bioprostheses and to reveal differences in local flow velocity, WSS and EL between the prosthesis types using 4D flow MRI at 1 year after implantation.

## Methods

### Study population

**Fig. 1:**
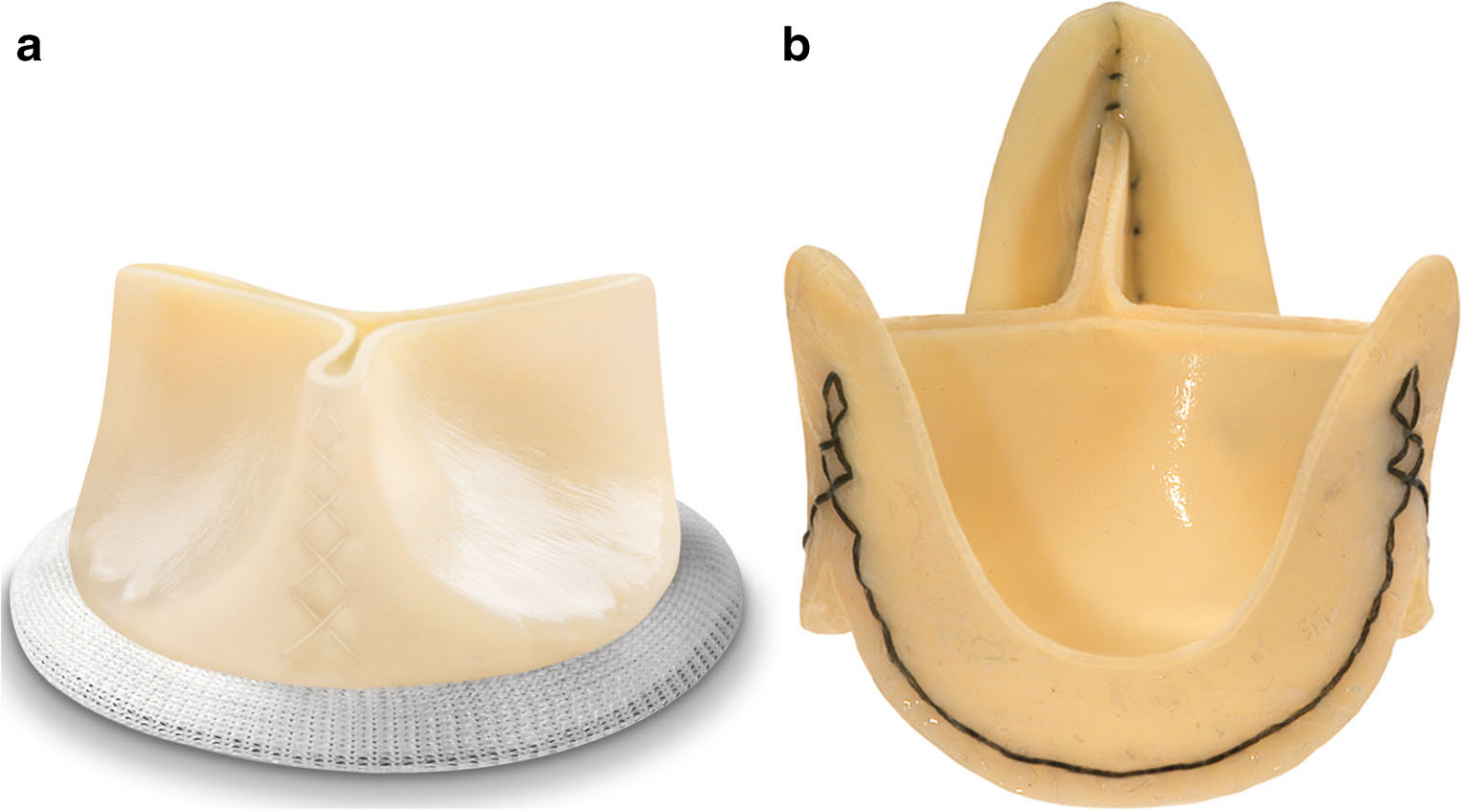
Example of the stented and stentless bioprosthesis. (**a**) Mitroflow prosthesis (Sorin, Saluggia, Italy); (**b**) Freedom Solo prosthesis (Sorin, Saluggia, Italy)

The Institutional Ethics Committee gave their approval for this exploratory cross-sectional study. All consecutive patients who underwent surgical aortic valve replacement within the previous 9–15 months were screened for exclusion criteria. In addition to standard MRI exclusion criteria, patients with a history of multiple heart valve replacements or known persistent atrial fibrillation were excluded. All eligible patients were asked to participate. After informed consent the MRI exam was conducted in 30 patients with either a stented Mitroflow (Sorin, Saluggia, Italy; Fig. 1a) or stentless Freedom Solo (Sorin, Saluggia, Italy; Fig. **1**b) bioprosthesis.

### MRI

All participants underwent a cardiovascular MRI at 1.5-Tesla (Magnetom Avanto, Siemens Medical Systems, Erlangen, Germany; software version B17). No contrast agent was used. The examination included a standard-of-care prospective electrocardiogram triggered and respiratory-gated short axis time-resolved (CINE) MRI for the evaluation of cardiac dimensions and function. For the assessment of aortic blood flow, time-resolved 3D phase-contrast MRI with three-directional flow encoding (4D flow) was obtained in a sagittal oblique 3D volume covering the aortic valve bioprosthesis and the thoracic aorta using electrocardiogram gating during free breathing with a respiratory navigator placed at the lung-liver interface. Pulse sequence parameters were as follows: echo time/pulse repetition time = 2.5/5.0 ms; bandwidth = 440 Hz/pixel; flip angle α = 7°; acceleration mode GRAPPA factor 2 with 24 reference lines, temporal resolution = 40 ms (18 phases); field of view (FOV) = 320 mm; spatial resolution 2.0 × 1.7 × 2.2 mm^3^. Velocity sensitivity was adjusted to minimize velocity aliasing (venc = 150–250 m/s), based on two-dimensional (2D)-flow measurements.

### Data analysis

Pre-processing included correction for Maxwell phase effects, eddy currents, velocity aliasing and noise masking using home-built Matlab software (Mathworks, Natick, MA, USA) [12]. For each time frame, a 3D volume of phase contrast magnetic resonance angiography (PC-MRA) data was created by multiplication of the phase contrast magnitude data with the absolute velocity images. The time-resolved PC-MRA volumes were subsequently averaged over all time frames. A commercial software package (MIMICS, Materialise, Leuven, Belgium) was used to create a 3D mask of the ascending aorta (from aortic annulus to brachiocephalic artery) by segmentation of the time-averaged PC-MRA data. Matlab Software was used to perform further post-processing.

First, the peak systolic time frame was determined by isolating the time frame with the highest velocity averaged over the segmentation. To reduce the effect of noise, the velocity field at peak systole was filtered with a 3 × 3 × 3 median filter. Peak velocity of the velocity field was determined using manual delineation of a region of interest (ROI) in the ascending aorta following a recently proposed methodology [13]. Furthermore, velocity magnitude at peak systole in the segmentation was averaged to yield mean velocity. Second, WSS was calculated by a previously published methodology [14]. In short, the axis system was rotated such that the z-axis aligned with the normal vector on a point on the vessel wall. A spline was fitted through three equidistant points along the inward normal (with length equals radius) containing the x-velocity values, with the velocity assumed to be zero on the first point at the wall. This was repeated for the y-velocities. By rotating the axis system back to the original system and multiplication with viscosity, the 3D WSS vector is obtained. This process was repeated for all points on the wall, resulting in a 3D WSS map of the ascending aorta. Third, EL due to viscous dissipation was calculated with the methods proposed by Barker et al. [8]. Briefly, viscous dissipation was calculated from the first-order spatial gradients of the 3D velocity field followed by summation over the segmentation and multiplication with viscosity. For both the WSS and EL calculation, a viscosity of 3.2 cP was assumed.

Two types of comparisons between the between-peak systolic haemodynamics for stented versus stentless valves were performed: (i) conventional comparison of peak and mean velocity, WSS and EL values averaged over the ascending aorta resulting in a median number per parameter (velocity, WSS, EL); (ii) comparison of local differences in 3D velocity, WSS and EL fields by cohort-averaging of the individual maps. In short, a shared ascending aortic geometry that represented all aortic shapes was generated using rigid registration for the combined stented and stentless cohorts [15]. Next, each individual aortic segmentation was registered with affine registration to the shared geometry followed by nearest neighbour interpolation of the individual 3D velocity, WSS and EL values to the shared geometry. After averaging the stented and stentless cohorts separately, cohort-averaged 3D maps for velocity, WSS and EL were obtained and displayed for the stented and stentless group. The statistical approach of this local-differences analysis was performed with per-voxel analyses using P-value maps, which is described in more detail in the statistics section. For method 1, the aortic velocity maps were displayed as a maximum intensity projection (MIP) in the sagittal direction. For method 2, cohort-averaged velocity and EL maps were displayed as a sagittal MIP, whereas cohort-averaged WSS maps were displayed as 3D renderings.

### Statistical analysis

For this exploratory study no sample size calculation was performed. Results were expressed as mean ± standard deviation, median with interquartile ranges or as number and percentile, as appropriate. Student’s t-test, Mann-Whitney U test and χ^2^ test were used to analyse differences between groups. For the conventional comparison of the averaged values of method 1, statistical analyses were performed using IBM SPSS statistics, version 22 (IBM Corp., Armonk, NY, USA).

For comparison of the local differences (method 2), p-value maps were created to investigate local significant differences in velocity, WSS and EL between the two cohorts with a methodology as previously described [15]. All subjects were registered, and the velocity, WSS and EL values were interpolated to the shared geometry. Next, a Mann-Whitney U test was performed between the stented and stentless cohort in each voxel containing a velocity and EL value and on each point on the wall containing a WSS value. The p-value maps for velocity, WSS and EL were displayed as 3D renderings. The volume of significance for velocity and EL was expressed as a percentage of the total volume. For WSS, the wall surface of significance was expressed as a percentage of the total wall surface. For all analyses a *p*-value < 0.05 was considered significant.

## Results

Of the 30 included patients, two patients were excluded: one due to claustrophobia and one because of poor quality of the 4D flow dataset due to paroxysmal atrial fibrillation. Of the 28 remaining patients, 14 had stented prostheses and 14 had stentless prostheses. The MRI examination took place 12 ± 1 months after aortic valve replacement for patients with stented prostheses and 13 ± 2 months for patients with stentless prostheses. Patients with a stented prosthesis less frequently had a concomitant procedure (21% vs. 71%, p = <0.01). Maximum ascending aorta diameters, left ventricle function and all other baseline characteristics were comparable (Table 1). At time of the MRI all patients were in New York Heart Association class 1.

**Table 1:** Patient characteristics

	Stented prosthesis	Stentless prosthesis	*p*-*value*
N	14	14	
Age (y)	74 ± 4	74 ± 6	0.89
Male	9 (64)	9 (64)	1.00
Time after operation (months)	12 ± 1	13 ± 2	0.21
Valve size distribution			0.91
21 mm	3 (21)	2 (14)	
23 mm	6 (43)	6 (43)	
25 mm	4 (29)	4 (29)	
27 mm	1 (7)	2 (14)	
Concomitant procedures	3 (21)	10 (71)	0.01
CABG	3	9	
MVP+TVP	0	1	
Baseline CMR measurements			
Max. diameter ascending aorta (mm)	36 (32-40)	37 (35-38)	0.67
LVEF (%)	64 (57-75)	61 (52-69)	0.25
Stroke volume (ml)	89 (75-105)	85 (69-111)	0.75
LVEDV (ml)	130 (124-142)	149 (122-182)	0.18
LDEDV corrected for BSA (ml/m^2^)	72 (64-81)	77 (67-87)	0.31
LVESV (ml)	45 (34-57)	50 (43-77)	0.17

*CABG* coronary artery bypass graft, *MVP* mitral valve repair, *TVP* tricuspid valve repair, *LVEF* left ventricular ejection fraction, *LVEDV* left ventricular end diastolic volume, *corrected LVEDV* LVEDV corrected for body surface area, *LVESV* left ventricular end-systolic volume

### Conventional averaged value analysis

**Fig. 2:**
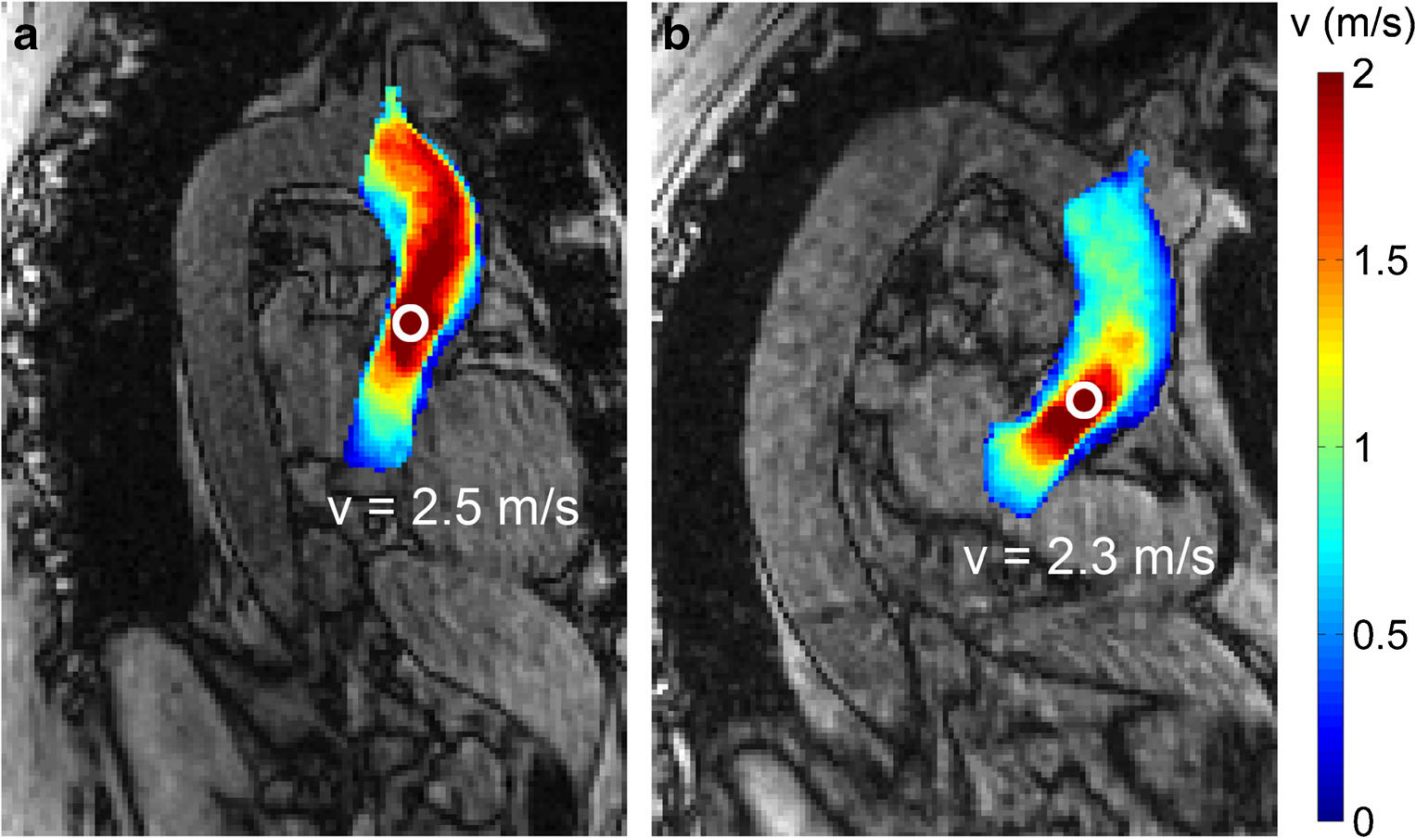
Example of velocity as measured with 4D flow MRI in the ascending aorta in a patient with (**a**) a stented prosthesis and (**b**) a stentless prosthesis. 0 = centre of peak velocity

Representative examples of velocity in the ascending aorta for a patient with a stented and a stentless prosthesis are displayed in an MIP in Fig. 2. Results of the measurement of averaged values over the ascending aorta are listed in Table 2. No statistically significant differences were found between the two prosthesis types in peak and mean velocity (stented vs. stentless median: 2.45 m/s vs. 2.11 m/s (p = 0.009) and 0.60 m/s vs. 0.62 m/s (p = 0.89), respectively) WSS (0.60 Pa vs. 0.59 Pa (p = 0.55) and EL (10.17 mW vs. 7.82 mW (p = 0.10).

**Table 2:** Four-dimensional flow cardiovascular magnetic resonance parameters in the ascending aorta 1 year after aortic valve replacement

Parameter	Stented prosthesis	Stentless prosthesis	*p*–*value*
N	14	14	
Peak velocity (m/s) (median, IQR)	2.45 (2.06–2.73)	2.11 (1.84–2.61)	0.09
Mean velocity (m/s) (median, IQR)	0.60 (0.53–0.73)	0.62 (0.49–0.72)	0.89
Mean WSS (Pa) (median, IQR)	0.60 (0.50–0.81)	0.59 (0.45–0.79)	0.55
Energy loss (mW) (median, IQR)	10.17 (6.86–13.36)	7.82 (4.84–10.68)	0.10

*IQR* interquartile range, *WSS* wall shear stress

### Local analyses

**Fig. 3:**
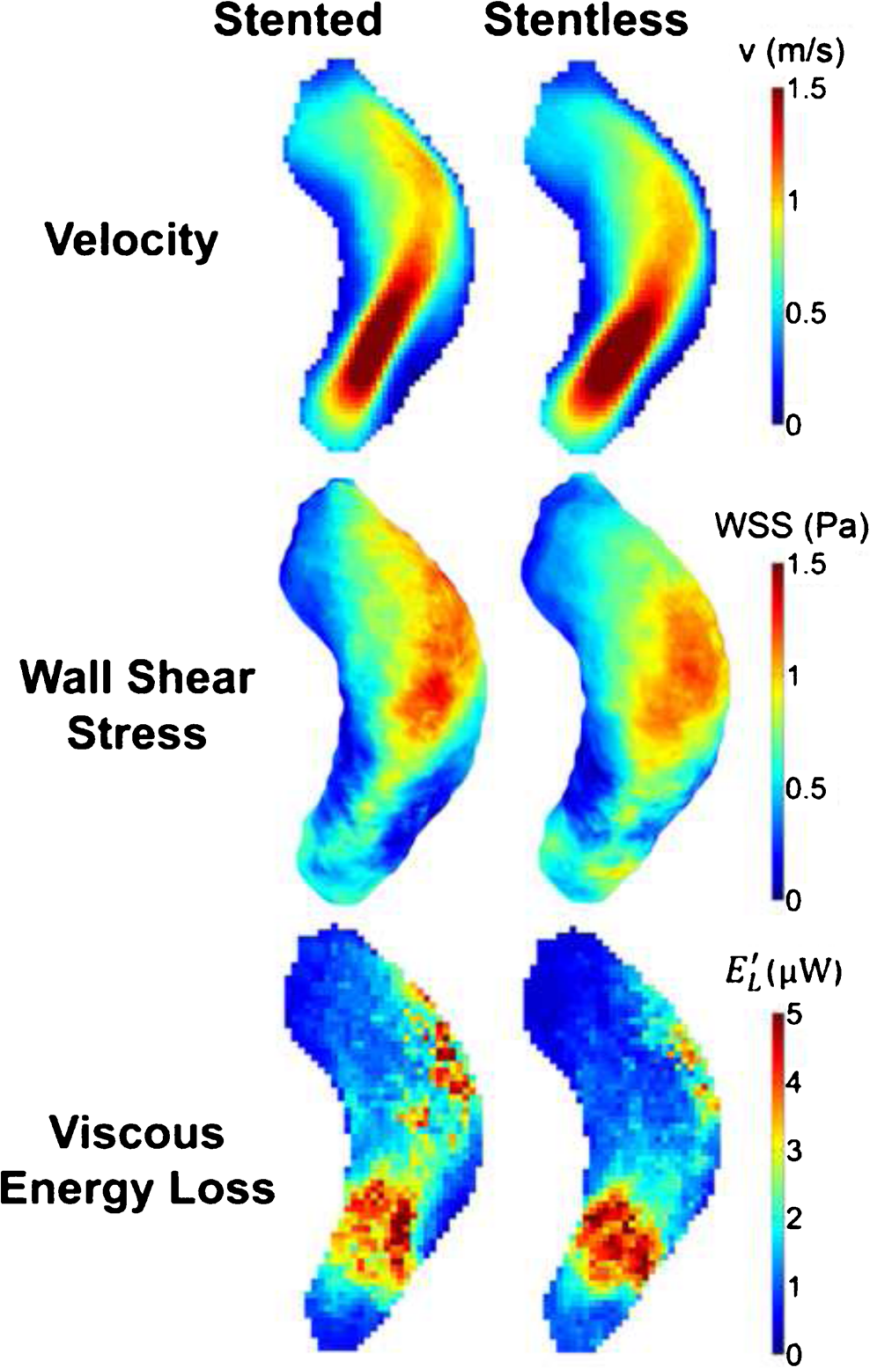
Cohort-averaged 3D maps for velocity, wall shear stress and viscous energy loss for the stented prosthesis (left column) and the stentless prosthesis (right column) displayed in a shared geometry

The qualitative differences between the prosthesis types are displayed in Fig. 3 in cohort-averaged 3D maps for velocity, wall shear stress and viscous energy loss for both prosthesis types. The averaged velocity jet stream for stentless prosthesis was more proximally located and a little broader as compared to the stented valves. Distal in the ascending aorta there was less WSS and EL in the map of patients with the stentless prostheses as compared to the stented prosthesis map.

**Fig. 4:**
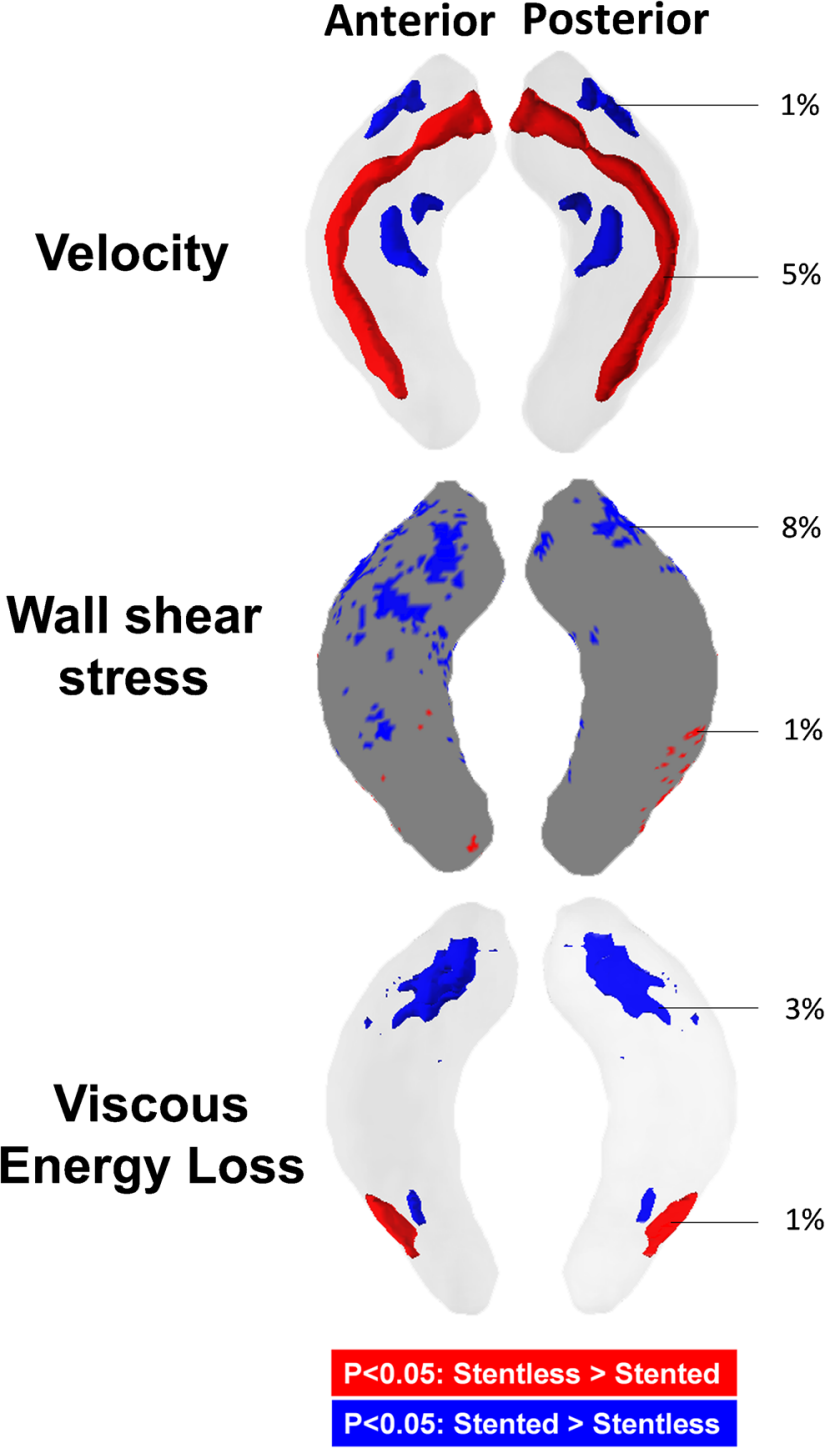
P-value maps displayed in a shared geometry of the ascending aorta from the anterior and posterior, displaying the significant differences for velocity, wall shear stress and viscous energy loss between stented and stentless prostheses. Red areas indicate significantly higher values for stentless prostheses and blue areas for stented prostheses. Numbers are cumulative percentages of the areas with significantly higher values as compared to the areas with no significant difference

For the statistical approach the corresponding p-value maps for the per-voxel analyses are displayed in a shared geometry in Fig. 4. At the sinotubular (ST)-junction level, velocity was comparable along the central lumen line for both prosthesis types. Over the distal descending aorta the map yields a higher velocity for stentless prosthesis with a profile mainly located in the central lumen. Significantly lower velocity was found at the outer lumen for the stentless cohort compared to the stented cohort. As displayed in Fig. 4 there was significantly higher velocity in 5% of the shared geometry for stentless and 1% for stented prostheses (Fig. 4). Additionally the maps displayed lower wall shear stress (1% vs. 8%) and lower viscous energy loss (1% vs. 3%) in the distal ascending aorta when compared to the stented prosthesis (Fig. 4).

## Discussion

By using 4D flow MRI we were able to visualize and quantify blood flow and flow-derived parameters of stented and stentless aortic valve bioprostheses in patients with similar baseline characteristics. In this study, 1 year after implantation, the velocity, WSS and EL of the stentless and stented aortic valve prosthesis were comparable when assessed for averaged values over the ascending aorta. Although not significant, there was a tendency towards lower peak velocities across the stentless prosthesis. The per-voxel analyses revealed a favourable flow profile with higher central lumen velocity but lower distal ascending aorta outer lumen velocity, WSS and EL in the stentless compared to the stented prosthesis. The higher central lumen velocity with a wider flow jet for the stentless prosthesis counter-intuitively indicates a flow profile with less obstruction or stenosis as compared to the flow profile caused by the stentless prosthesis. Please note that both prostheses remain associated with some degree of obstructions as can be interpreted by the measured peak velocities of more than 2 m/s [2]. Since no differences were found in the averaged values, we can conclude that these local differences averaged out over the ascending aorta. With these results we believe that this study contributes to the ongoing debate on the optimal prosthesis type for aortic valve replacement. Optimisation of the haemodynamic performance of the prosthesis is important. Obstructed blood flow over the prosthesis may lead to valve leaflet deterioration and potentially the need for re-intervention. In addition, optimal performance reduces the left ventricular workload to facilitate ventricular remodelling and thereby lowering myocardial mass. Reduction of the left ventricular mass is considered an indicator of successful aortic valve stenosis treatment as residual hypertrophy, or lack of remodelling, is associated with increased mortality [16, 17]. To optimise long-term outcome, the valve prostheses should provide unobstructed central laminar blood flow without substantial energy loss and shear stress on the aortic wall comparable to energy loss and shear stress on the native aortic valve. In our study, although the peak velocity presented is elevated and comparable to that of a phantom with severe stenosis, the native velocity, WSS and EL pattern was best resembled by the stentless prosthesis [18]. Additional evidence of the obstruction that is still present in both prostheses is provided by a comparison with results of 4D flow MRI of the ascending aorta of healthy volunteers in recent literature [8, 19]. Energy loss of patients in our study was noticeably higher compared to (younger) healthy controls (12 controls, energy loss 1.2 mW) and was more comparable with patients with aortic valve stenosis and a dilated aorta (14 patients, energy loss 10. 9 mW) [8]. In addition, peak velocity, mean velocity and wall shear stress were considerably higher in our patients compared to the healthy volunteers of the same age (peak velocity 1.50 m/s, mean velocity 0.46 m/s, wall shear stress 0.48 Pa) [19]. Please note that the differences in velocity, WSS and EL between our patients and the literature cannot be attributed to aortic diameters, as the diameters of the ascending aortic in our patients were similar to reference values of men and women of a comparable age [20]. Therefore we believe that to achieve a haemodynamic performance that is comparable to that of the native aortic valve, there is still a long way to go in the further development and improvement of aortic valve bioprostheses.

Since the development of stentless bioprostheses, it is believed that this type of prosthesis mimics the native flow pattern better than a stented prosthesis. The absence of the stent should contribute to lower velocity, wall shear stress and energy loss of the valve. However, previous echocardiography studies did not display haemodynamic benefit in terms of left ventricular mass regression or postoperative mean gradients [5, 21]. Furthermore, we did not find a difference in the averaged peak systolic velocities. Von Knobelsdorff-Brenkenhoff and colleagues, who used 4D flow MRI, demonstrated differences in vorticity, helicity and eccentricity among a heterogeneous group of controls and patients with autografts, mechanical, stentless and stented aortic valve prostheses [4]. Their comparison of eight patients with a stented bioprosthesis with 14 patients with a stentless bioprosthesis revealed that stentless prostheses were associated with larger effective orifice areas and less vorticity and helicity [4]. This is in agreement with our per-voxel analysis that yielded higher central lumen flow velocities in the stentless group, which implies the occurrence of less vorticity and helicity. However, a per-voxel analysis was not performed in their study, inhibiting further comparison of data [4].This study has limitations, mainly related to the small sample size as a consequence of the study design. We describe a tendency towards lower peak velocities across the stentless prosthesis. A larger sample size could have influenced those results. However, for this exploratory study we did not use sample size calculation prior to scanning. Future studies should address this comparison of peak velocities further. In addition, our 4D flow MRI data were acquired without the use of intravenous contrast media. Although the signal-to-noise ratio in our data could be improved by the use of intravenous contrast, we found image quality to be sufficient for analysis. Additionally, quantification of 3D wall shear stress and viscous energy loss depend on spatial resolution [14, 22]. By ensuring identical spatial resolution for all scans, comparison of wall shear stress and viscous energy loss between valve types was possible.

Currently, longitudinal follow-up data of 4D flow MRI parameters are missing. Future studies are necessary to acquire comprehensive advanced baseline MRI data with long-term follow-up of enrolled patients. Ideally, 4D flow MRI will be combined with sequences for tissue characterization and clinical outcome of different aortic valve prostheses to determine prognostic parameters for individual outcome and patient specific risk stratification.

## Conclusion

Stented and stentless aortic valve prostheses exhibit comparable flow velocity and wall shear stress when assessed for averaged values in the ascending aorta. However, for stentless prostheses the local flow profiles described with a per-voxel analysis revealed a less obstructed profile more similar to the native aortic valve with a significantly higher central velocity profile and lower values for outer lumen velocity, wall shear stress and energy loss.

## Abbreviations

4D: Four-dimensional
3D: Three-dimensional
MRI: Magnetic resonance imaging
